# CK2 blockade causes MPNST cell apoptosis and promotes degradation of β-catenin

**DOI:** 10.18632/oncotarget.10668

**Published:** 2016-07-18

**Authors:** Jed J. Kendall, Katherine E. Chaney, Ami V. Patel, Tilat A. Rizvi, David A. Largaespada, Nancy Ratner

**Affiliations:** ^1^ Division of Experimental Hematology and Cancer Biology, Cincinnati Children's Hospital, Department of Pediatrics, University of Cincinnati, Cincinnati, OH 45229, USA; ^2^ Department of Pediatrics, Masonic Cancer Center, University of Minnesota, Minneapolis, MN 55455, USA

**Keywords:** MPNST, CK2, G2 arrest, NF1, β-catenin

## Abstract

Malignant peripheral nerve sheath tumors (MPNSTs) are soft tissue sarcomas that are a major cause of mortality of Neurofibromatosis type 1 (NF1) patients. MPNST patients have few therapeutic options available and only complete surgical resection can be curative. MPNST formation and survival are dependent on activated β-catenin signaling. The goal of this study was to determine if inhibition of the CK2 enzyme can be therapeutically exploited in MPNSTs, given CK2's role in mainta ining oncogenic phenotypes including stabilization of β-catenin. We found that CK2α is over-expressed in MPNSTs and is critical for maintaining cell survival, as the CK2 inhibitor, CX-4945 (Silmitasertib), and shRNA targeting CK2α each significantly reduce MPNST cell viability. These effects were preceded by loss of critical signaling pathways in MPNSTs, including destabilization of β-catenin and TCF8. CX-4945 administration *in vivo* slowed tumor growth and extends survival time. We conclude that CK2 inhibition is a promising approach to blocking β-catenin in MPNST cells, although combinatorial therapies may be required for maximal efficacy.

## INTRODUCTION

Half of MPNSTs arise in context of NF1 disease, typically within benign plexiform neurofibromas [1], while the other half arise sporadically with no obvious precursor lesion or disease [2, 3]. The lifetime risk for NF1 patients developing a MPNST is 8–13%. The 5-year survival rate for patients with a sporadic or NF1 derived MPNST is poor (42% and 21%, respectively) [3]. Surgical resection is the standard treatment for MPNSTs, but is often ineffective due to high rates of local tumor recurrence, distant metastasis and/or the site of the lesion precluding complete resection. If resection is incomplete, the patient 5-year survival rate is usually not impacted by therapies such as radiation or chemotherapy [4, 5].

Studies suggest that MPNST tumorigenesis is initiated when cells of the Schwann lineage lose expression of the *NF1* gene which encodes neurofibromin, a RAS-GAP protein; loss of neurofibromin delays RAS-GTP hydrolysis [6–10]. Progression to malignancy is associated with additional mutations in the NF1 null cells within the Schwann lineage, most commonly biallelic loss of *CDKN2A* [11, 12]; mutations in PTEN, p53, and RB pathway genes have also been observed. [13–15].

Therapeutic strides against MPNSTs have been made in the pre-clinical setting. Drugs targeting MEK, a downstream effector of RAS, slow MPNST growth in pre-clinical mouse models [16–18]. Aurora-A-Kinase inhibitors have also been shown to cause prolonged growth arrest in preclinical mouse models [19]. To date, no single agent has been shown to be curative in animal models or in the clinic. Combinations of agents are showing more promise [20–23], and trials are being initiated to test their efficacy.

Understanding fundamental and unique survival mechanisms of MPNSTs should enable new therapeutic approaches. β-catenin is believed to be a critical regulator of MPNST survival. Over-expression of β-catenin in immortalized human Schwann cell lines (iHSCs) causes features of malignant transformation, and knockdown of β-catenin decreases MPNST cell viability [24–26]. Therefore, it is believed that targeting β-catenin might be a useful therapeutic strategy in MPNST. Indeed, targeting β-catenin is an attractive potential therapy in many cancer types [27]. While β-catenin is largely cytoplasmic in normal Schwann cells. However, in both neurofibromas and MPNSTs β-catenin shows pronounced nuclear localization leading to transcriptional activation of target genes [24, 25, 28]. β-catenin is degraded by a complex involving GSK-3β, AXIN1, APC, CK1 and others that dock to β-catenin [29–33]. Once docked to the destruction complex, β-catenin is phosphorylated by GSK-3β and CK1. This phosphorylation serves as a marker for ubiquitination and ultimately, degradation by the proteasome [34–36]. Mutations in β-catenin destruction complex genes lead to various cancers due to abnormally high levels of stabilized β-catenin protein [37–40]. However, targeting components of the canonical β-catenin destruction complex has to date been unsuccessful. It may be that targeting β-catenin without causing toxicity will require finding targets unique to a cancer that do not directly impact the ubiquitous GSK-3β destruction complex.

Most cancers share hallmark characteristics including hyper-proliferation and evasion of Programmed cell death that are often governed by similar molecular mechanisms [41]. For example, Casein Kinase 2 (CK2) is a serine/threonine kinase that is a regulator of oncogenic processes in diverse cancers [42, 43]. Some of the pro-growth and survival pathways regulated by CK2 include WNT/β-catenin, Erk, and AKT signaling [44–49]. CK2 promotes β-catenin signaling by phosphorylating β-catenin in the armadillo arm region, preventing docking with the GSK-3β destruction complex [44]. CK2 also negatively regulates growth inhibitory pathways involving PTEN [50, 47, 51], suppresses apoptosis [52–55], and phosphorylates caspase 9 preventing cleavage from caspase 8 and evading cell Programmed apoptosis [56]. CK2 exists as a holoenzyme containing α and β subunits [42, 57]. In cancer, CK2α is often overexpressed [58, 59] creating the formation of catalytically active CK2α dimers that are able to phosphorylate targets unrecognized by the CK2α/ CK2β holoenzyme [60]. A defining characteristic of CK2α dimers is constitutive activity. There are no known negative feedback loops to inhibit CK2 signaling. This unrestrained activation of downstream targets may be a key facilitator of oncogenic processes.

A selective CK2 inhibitor, CX-4945, developed by Cylene pharmaceuticals, is currently in phase II clinical trials as an anti-tumor agent [61, 62]. Using CK2 inhibitors alone or in combination with other drugs that target overlapping oncogenic pathways may be a useful therapeutic strategy. CK2 also plays a role in DNA damage repair. Therefore, CX-4945 is currently being tested in combination with Gemcitibene and Cisplatin in non-resectable cholangiocarcinomas [63, 64, 54].

In this study, we demonstrate that CK2α is over-expressed in MPNST and is an essential regulator of MPNST survival. We show that pharmacological or genetic inhibition of CK2 diminishes MPNST cell viability and that CX-4945 slows tumor growth *in vivo*, extending the survival of mice with MPNST xenografted tumors. The mechanism by which CK2 facilitates MPNST survival is multifactorial, as CK2 inhibition decreases β-catenin and TCF-8 protein stability, causes cell cycle arrest, and induces apoptosis.

## RESULTS

### CK2 is overexpressed in MPNSTs

**Figure 1:**
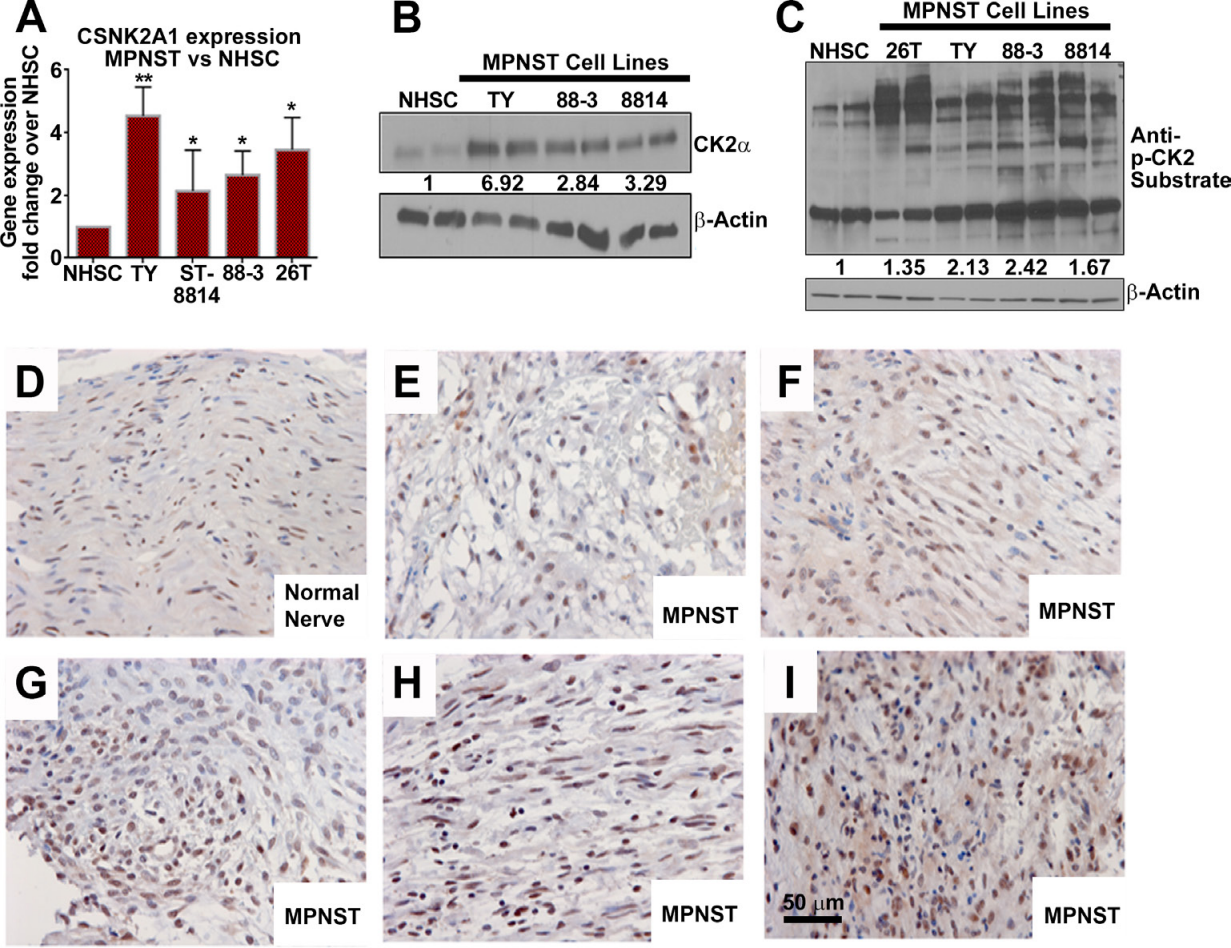
(**A**) Three designated MPNST cell lines show over-expression of *CSNK2A1* mRNA vs NHSCs. (**B**) Western blot analysis shows that CK2α protein is overexpressed in MPNST cell lines vs NHSCs. (**C**) Western blot analysis using an antibody that detects CK2 substrate phosphorylation reveals increased CK2 activity in MPNST cell lines. Some CK2 targets are phosphorylated in MPNSTs but not in NHSCs. (**D**–**I**) Expression of CK2α shown by IHC in human nerve (**D**) and MPNST patient biopsy samples (**E**–**I**). Asterisks in A indicate statistically significant differences (*p < 0.05, ** *p* < 0.01, ***p < 0.001). QPCR results are shown as the mean ± standard deviation (S.D.) of three independent biological replicates, each in triplicate. Western blots are representative of at least 3 independent experiments.

We measured CK2α protein and CK2α mRNA expression in MPNST cell lines. QPCR revealed higher CK2α mRNA expression in MPNST cell lines (2 to 5 fold) fold as compared to control normal human Schwann cells (NHSCs) (Figure 1A). The CK2α protein was also overexpressed in each MPNST cell line (Figure 1B). If CK2α is important for MPNST survival, then overexpression of CK2α should lead to abnormal phosphorylation patterns of CK2 substrates. Western blot analysis of MPNST cell lines versus NHSCs using an anti-CK2 substrate antibody (which recognizes CK2 consensus target motif pS/pTDXE only when phosphorylated) shows that CK2α overexpression correlates with increased phosphorylation of CK2 target proteins. We identified CK2 substrate phosphorylation bands unique to MPNSTs based on size observed on the western blot. The identity of these bands is unknown to date (Figure 1C). CK2α expression was analyzed in multiple human MPNST tissue (*n* = 6 different patients) and nerve (*n* = 2) biopsies using immunohistochemistry (IHC). CK2α immunoreactivity appeared increased in MPNSTs as compared to the normal nerve, but showed variability among the MPNST samples (Figure 1D–1I).

### CK2 inhibition induces cell death and cell cycle arrest in MPNSTs *in vitro*

**Figure 2:**
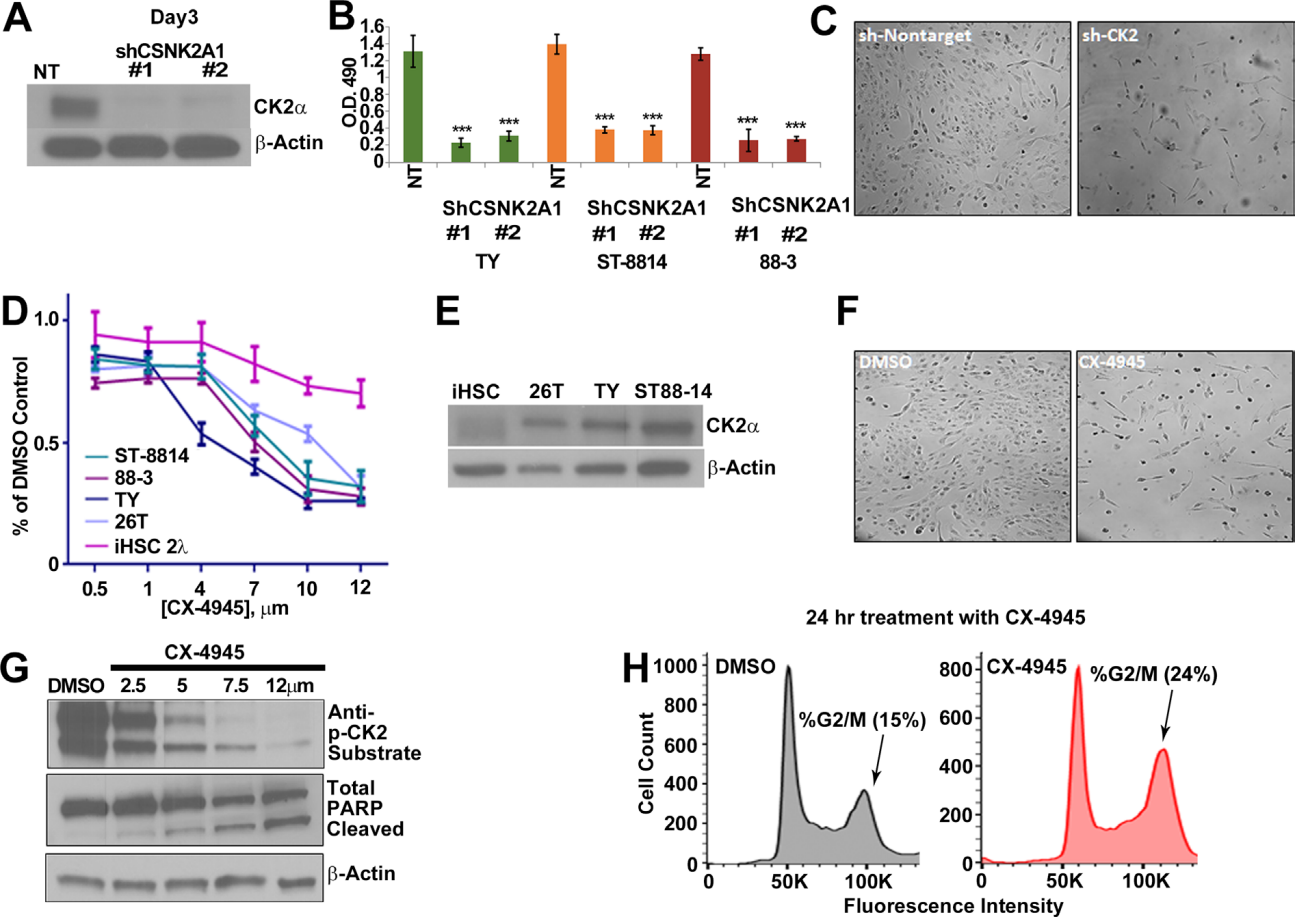
(**A**) MPNST (S462-TY) cells were treated with 2 unique shRNAs targeting CSNK2A1 for 72 h, and western blot analysis confirmed a reduction of CK2α protein. NT = cells treated with a non-targeting shRNA control. (**B**) Three day CK2α shRNA treatment had a cytotoxic effect on MPNST cell lines as measured by MTS; y-axis is O.D. reading at 490 nm. (**C**) Phase contrast photomicrographs show S-462TY cells 3 days after NT or shCK2α #1. (**D**) Increasing concentrations of CX-4945 (72 h) decrease MPNST cell line growth. CX-4945 has less effect on control iHSC cells as measured by MTS; y-axis is O.D. reading at 490 nm. (**E**) CK2α is overexpressed in MPNST cell lines as compared to the iHSC control cell line. (**F**) Phase contrast shows that CX-4945 treatment depletes cell population at 24 h as compared to the DMSO control. (**G**) MPNST cell lines show a decrease in CK2 activity in response to escalating CX-4945 concentrations (24 h) as measured by a western blot analysis using anti-CK2 substrate, and to undergo apoptosis as indicated by increased cleaved PARP. H. CX-4945 causes MPNST cells (S462-TY) to increase the percent of cells in the G2/M phase of the cell cycle as indicated by flow analysis of propidium iodide stained cells (S.D. of DMSO = 1.8% and CX4945 = 3.8%). Asterisks in B indicate differences after Student's *t*-test (*p < 0.05, **p < 0.01, ***p < 0.001). CX-4945 cell viability assay, MTS, and qPCR results are the mean of three independent biological replicates each in triplicate, ± S.D.

To test if MPNST cells depend on CK2α for survival, MPNST cell lines were treated with two different CSNK2A1 shRNAs, each of which decreased expression of the CK2α protein (Figure 2A). MPNST cell viability, as measured by MTS assay, was significantly reduced by the knockdown of CK2α at 72 h post infection (Figure 2B, 2C). We confirmed the sh-CSNK2A1 knockdown results by the pharmacological inhibition of CK2. CX-4945 is an anti-tumor agent in phase II clinical trials that inhibits CK2 activity by competitively binding to the CK2 enzyme active site [65]. To determine the effective concentrations of CX-4945, MPNST and iHSC cell lines were treated with CX-4945 for 72 h and cell viability was measured through MTS absorbance. At approximately 6 μm CX-4945 decreased NF1 derived MPNST cell line viability by 50%. CX-4945 at 10 μm had maximal effect on NF1 derived MPNST cell line survival, causing significant reduction of viability at 72 h. The 26T sporadic MPNST cell line was more resistant to CX-4945 than the NF1 derived MPNST cell lines. An immortalized human Schwann cell (iHSC) control cell line was significantly more resistant to CX-4945 treatments as compared to MPNST cell lines (Figure 2D, 2E). MPNST cell lines were treated with CX-4945 for 24 h and harvested for western blot analysis. The antibody against CK2 phosphorylated target motif pS/pTDXE showed that increasing concentrations of CX-4945 progressively decreased CK2 substrate phosphorylation (Figure 2F). CX-4945 treatment induced apoptosis as indicated by increased cleaved PARP (Figure 2F) and increased the percent of cells in the G2/M phase of the cell cycle, an indicator of cell cycle of arrest (Figure 2G).

### CK2 regulates β-catenin protein stability in MPNSTs *in vitro*

**Figure 3:**
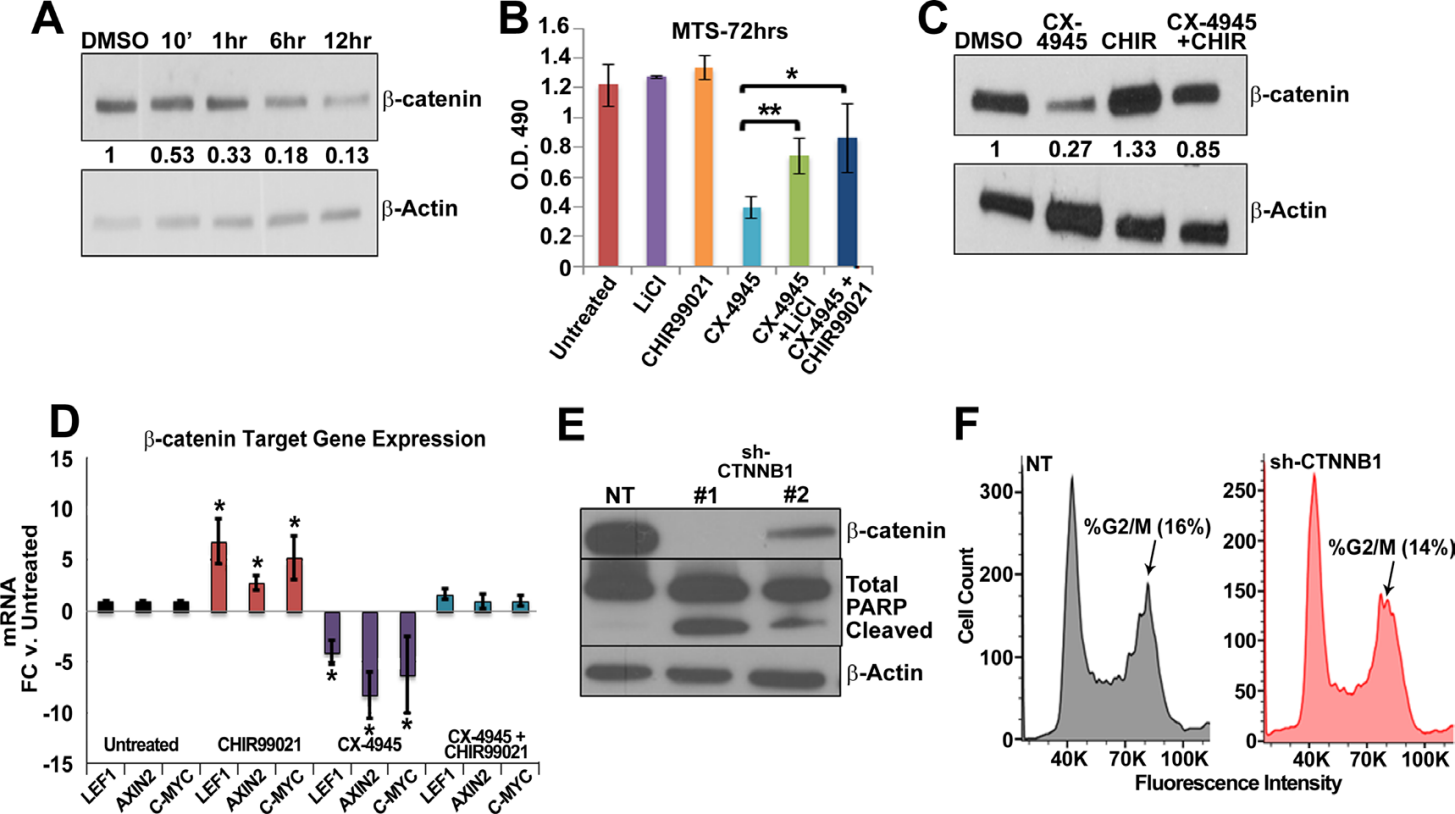
(**A**) Time course treatment of CX-4945 at 10 μm decreases β Catenin protein as early as 10 min in western blot analysis in MPNST (S462-TY) cells. (**B**) Partial rescue of CX-4945 MPNST cytotoxicity is achieved by inhibiting GSK-3β through CHIR99021 or LiCl as measured through MTS assay 72 h. post treatment; y-axis is O.D. reading at 490 nm. (**C**) Treatment with GSK-3β inhibitors restored β Catenin protein in CX-4945 treated MPNST cells (24 h) as measured by western blot analysis. (**D**) Treatment with GSK-3β inhibitors restored β-Catenin target gene mRNA expression in CX-4945 treated MPNST cells (24 h) as measured by qPCR. (**E**) Western blot shows that shCTNNB1 induces apoptosis (cleaved PARP) in MPNST cells (S462-TY). (**F**) MPNST (S462-TY) cells treated with shRNA containing a non-targeting sequence or against β-Catenin and then stained with propidium iodide. Flow analysis of these samples reveals no significant difference in the cell cycle profile. (S.D. of NT = 1.6% and shCTNNB1 = 5.3%). Western blot above the cell cycle analysis shows that the shRNA effectively targeted β-Catenin. Asterisks indicate statistically significant differences (*p < 0.05, **p < 0.01, ***p < 0.001); Student's *t*-test. MTS and qPCR results are the mean of three independent biological replicates each in triplicate ± S.D.

CK2 activity can induce canonical WNT signaling pathway activation by phosphorylating β-catenin in its armadillo repeat region [44]. This phosphorylation prevents interaction with the GSK-3β destruction complex, allowing β-catenin to translocate to the nucleus and induce target gene transcription [44, 66]. As β-catenin expression is important for MPNST growth and survival [24, 25], and CK2 is elevated in MPSNTs, we hypothesized that CK2 might regulate β-catenin protein stability. CX-4945 treatment reduced β-catenin protein levels by 10 min. and caused profound reduction by 12 h (Figure 3A). MPNST treatment with CX-4945 did not affect CTNNB1 mRNA levels (data not shown). This data suggests that CK2 likely regulates β-catenin protein stability in MPNST cells through a protective phosphorylation of the armadillo repeat region. Loss of β-catenin decreased MPNST cell viability in previous studies [24, 25]. To begin to test if stabilization of β-catenin protein could rescue the cytotoxic effects of CK2 inhibition, we treated MPNST cells with CX-4945 and GSK-3β inhibitors LiCl or CHIR92201. Treatment with GSK-3β inhibitors partially rescued the cytotoxicity of CK2 inhibition (Figure 3B), as β-catenin protein levels (Figure 3C) and downstream target gene expression AXIN2, LEF1, and C-MYC were restored (Figure 3D). To determine if β-catenin knock down phenocopies the G2/M arrest or apoptosis caused by CK2 inhibition, cells were treated with sh-CTNNB1 for 72 h and subjected to propidium iodide flow or western blot analysis. β-catenin knockdown phenocopied apoptosis as indicated by cleaved PARP (Figure 3E). However, shCTNNB1 did not phenocopy CK2 mediated changes in the cell cycle profile (Figure 3F) suggesting that CK2 regulates additional mechanisms of cell survival.

### CK2 regulates survival factor TCF8 in MPSNTs *in vitro*

**Figure 4:**
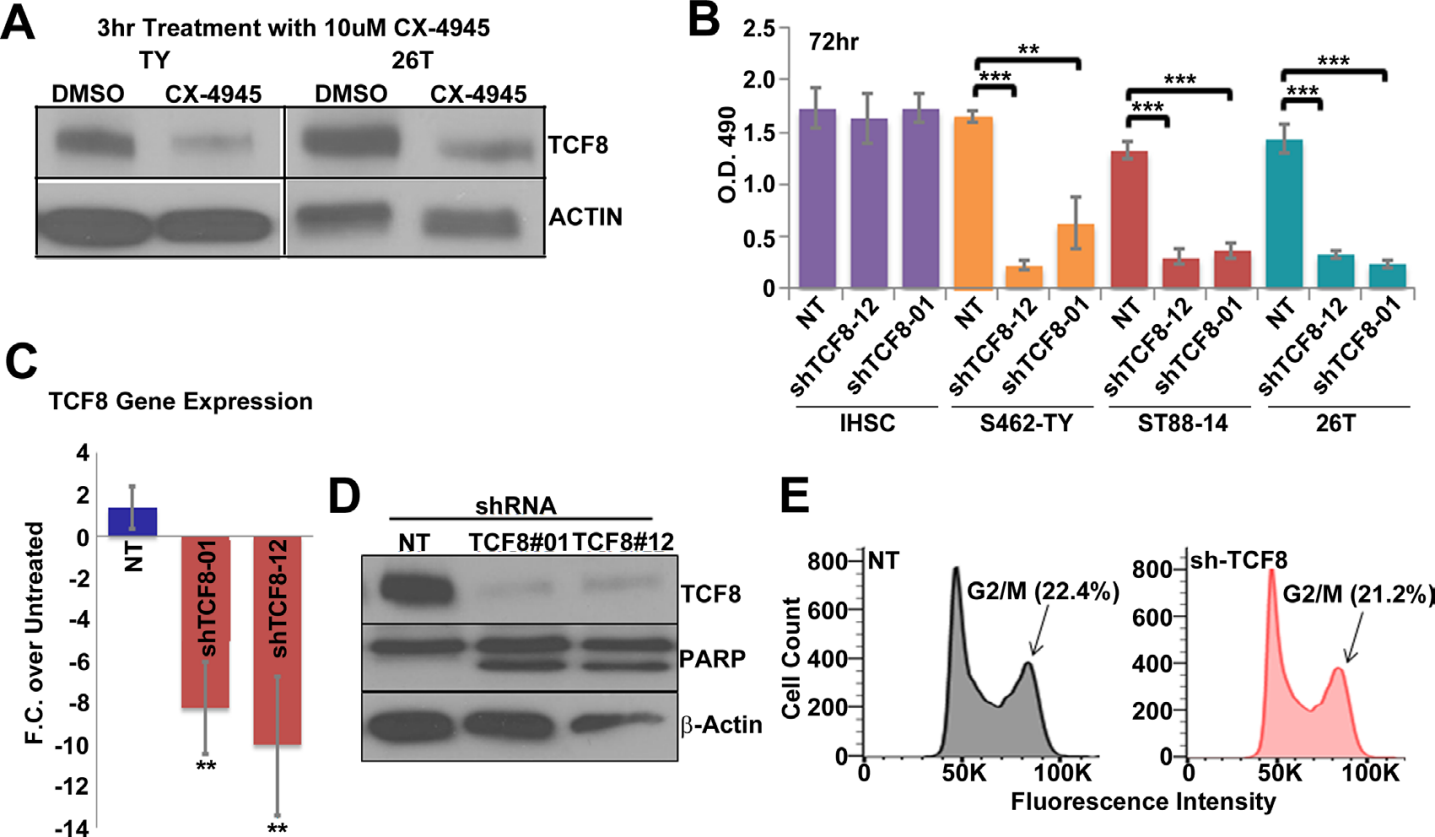
(**A**) CX-4945 treatment for 3 h decreases TCF8 protein expression in MPNST cell lines (S462-TY (TY) and 26T). (**B**) A 72 h. treatment with TCF8 shRNA caused a significant decrease in MPNST cell viability in multiple MPNST cell lines, but not iHSC, in an MTS assay. NT = cells treated with non-targeting shRNA control. (**C**) TCF8 shRNA decreased TCF8 RNA expression in MPNSTs (S462-TY) 72 h post treatment. (**D**) TCF8 shRNA decreased TCF8 protein levels and induced apoptosis in MPNST cells (S462-TY) as indicated by increased levels of cleaved PARP. (**E**) TCF8 knockdown did not alter cell cycle progression (S.D. of G2/M phase: NT = 1.7% and shTCF8 = 1.3%). Asterisks in B indicate statistically significant differences as for other figures. MTS, Flow, and qPCR results are the mean of three independent biological replicates in triplicate, ± S.D.

To investigate mechanisms of survival regulated by CK2 that are independent of β-catenin we overlapped lists of known and predicted CK2 targets to genes overexpressed in MPNSTs, resulting in three potential targets: SIX1, TWIST1, and TCF8 [67, 68]. Of the potential three targets only TCF8 decreased in response to CK2 inhibition (Figure 4A). Over-expression of the CK2α subunit has been shown to drive over-expression of TCF8 in breast cancer and TCF8 contains potential CK2 phosphorylation sites [68–70]. To test if TCF8 is involved in MPNST survival we treated MPNSTs with TCF8 specific shRNA. Treatment of MPNSTs with TCF8 shRNA significantly decreased cell viability but had little effect on iHSCs as measured by a MTS assay 3 days post infection (Figure 4B). We demonstrate that the TCF8 shRNA knocks down TCF8 mRNA and protein by day 3 (Figure 4C, 4D). MPNSTs treated with shTCF8 undergo apoptosis as demonstrated by cleaved PARP western blot analysis (Figure 4D). However, cell cycle progression was not perturbed in MPNSTs through shRNA knockdown of TCF8 (Figure 4E).

### CX-4945 slows tumor growth *in vivo*

**Figure 5:**
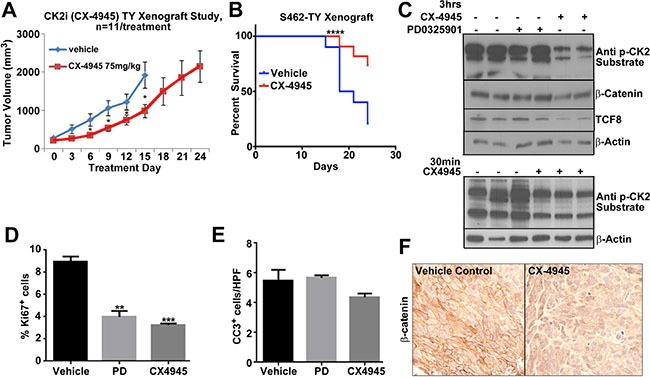
(**A**) MPNST xenograft growth was significantly reduced by CX-4945 (*p* < 0.05 = *). (**B**) Survival of mice was significantly increased by CX-4945 treatment (*p* < .0001 using a log-rank *Mandel-Cox* test). (**C**) Tumors were harvested from mice treated with CX-4945 at 30 min or 3 h post last dose. Western blot analysis indicates that CX-4945 inhibited CK2 substrate phosphorylation. β-catenin and TCF8 protein levels were also reduced at the 3 h time point. Top, PD0325901 did not affect these targets. (**D**) Tissue sections from MPNST xenografts harvested 3 h. after the last dose of CX-4945 show significantly decreased cell proliferation (Ki67+ cells). (**E**) Cell death (Cleaved Caspase 3) did not change in treatment groups (Cells per high-powered field; HPF). (**F**) β-catenin immunohistochemistry confirms that CX-4945 decreases β-catenin protein in MPNST xenografts. In E, F, effects of the MEK inhibitor are shown for comparison. Asterisks in D indicate statistically significant differences analyzed by ANOVA (*p < 0.05, **p < 0.01, ***p < 0.001).

To test the effectiveness of CX-4945 *in vivo*, athymic nude mice were injected with 1.5 million MPNST (S462-TY) cells. Treatment began when xenografts grew to an average of 250 MM3. The study continued for 24 days, or until tumor burden reached 2500 mm^3^. At a dose of 75 mg/kg twice daily, CX-4945 slowed MPNST tumor growth by approximately 50% through 15 days of treatment (Figure 5A). CX-4945 also significantly increased the survival of engrafted mice, with 66% of CX-4945 treated mice surviving through day 24, while only 20% of the vehicle control treated mice survived 24 days (Figure 5B). Tumors were harvested 30 min and 3 h post CX-4945 treatment, and substrates were analyzed. CX-4945 treatment decreased phosphorylation of CK2 targets at 30 min and 3 h. β-catenin and TCF8 protein levels were, however, only decreased at the 3 h time point (Figure 5C, 5F). CX-4945 also decreased Ki67 expression indicating reduced proliferation (Figure 5D), but did not induce apoptotic cell death as measured by cleavage of Caspase 3 in MPNST xenografts (Figure 5E).

### MPNST treatment with CX-4945 in combination with PD0325901

**Figure 6:**
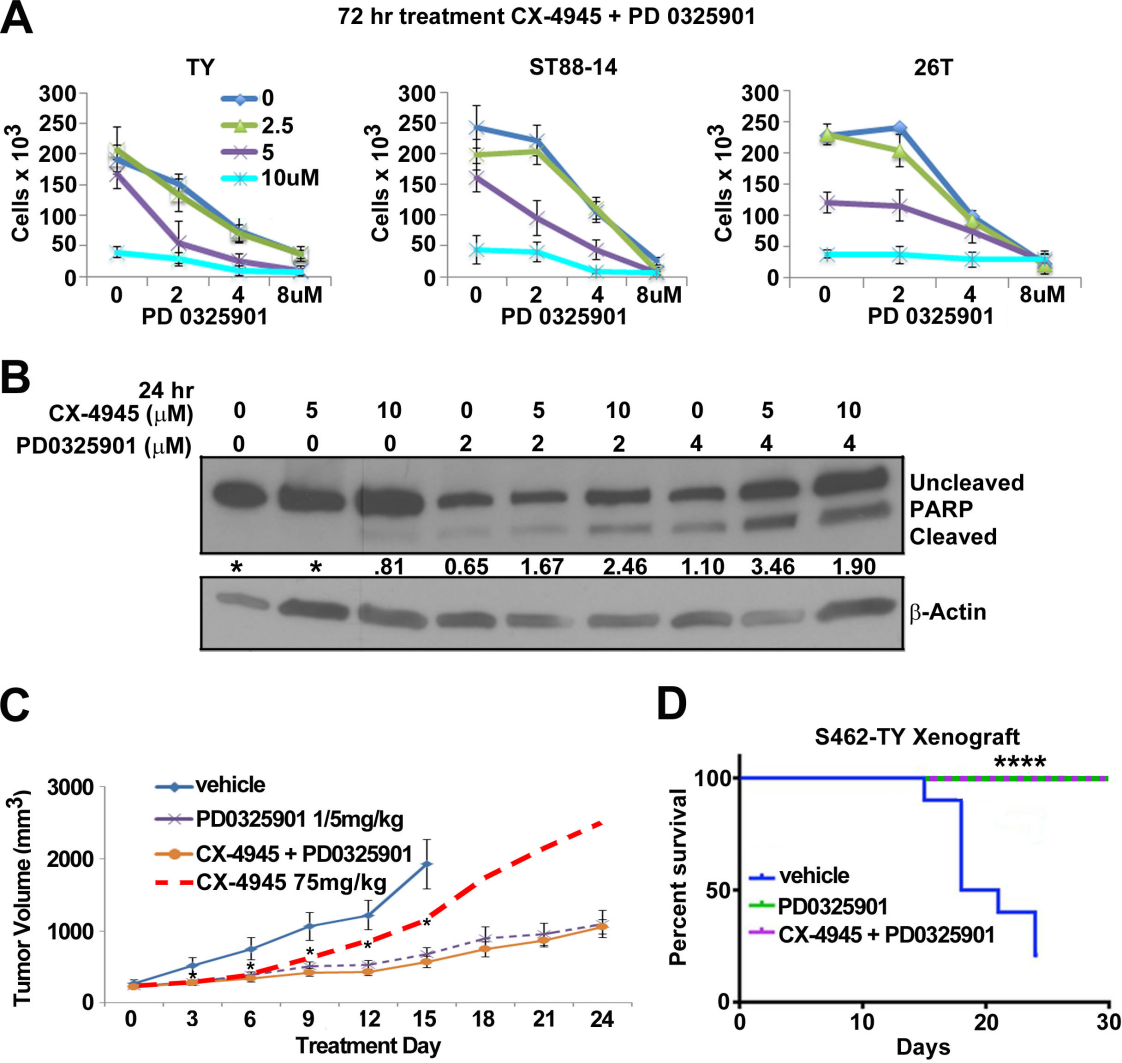
(**A**) 72 h combination treatment of CX-4945 and PD0325901 at concentrations 5 and 2 μm respectively had an additive to synergistic effect on MPNST cell lines S462TY and ST88-14, but little effect on the sporadic 26T cell line. (**B**) MPNST cells treated for 24 h with CX-4945, PD0325901, or the combination revealed increasing PARP cleavage in combination treatments. (**C**) S462TY cells were grafted into nu/nu mice and segregated into vehicle, CX-4945, PD0325901, or CX-4945 + PD0325901 groups and were treated for 24 days. CX-4945 and PD0325901 both delayed tumor growth, however the CX-4945 + PD0325901 treatment did not slow tumor growth more than the PD0325901 treatment alone. CX-4945 tumor growth results represented by the dashed redline (data shown in Figure 5A). (**D**) Mice were removed from the survival curve at time of death or when the tumor volume reached 2500 mm^3^. All mice treated with PD0325901 or CX-4945 + PD0325901 survived until the end of the study at Day 30 (*p* < .0001 using *log*-rank *Mandel-Cox* test). Asterisks in A ndicate statistically significant differences (*p < 0.05, **p < 0.01, ***p < 0.001). *In vitro* MTS results are the mean of three biological replicates ± S.D.

Hyper-activated RAS signaling is a hallmark of the NF1 syndrome. RAS activates downstream targets including MEK. Previous reports showed that CX-4945 can sensitize head and neck cancer to MEK inhibitors and MEK inhibition has been shown to slow MPNST tumor growth [71, 16]. Therefore, we hypothesized that combination treatment with a MEK and CK2 inhibitor might be a more effective MPNST treatment than either single agent alone. To test this idea MPNSTs were treated with CX-4945, PD0325901, or PD0325901 and CX-4945 *in vitro* and cells were counted after 72 h. Combination treatment decreased the MPNST cell population by 50–70% as compared to cells treated with either CX-4945 or PD0325901 alone and the combination index (C.I. = 0.85) suggests that CX4945 with PD0325901 is moderately synergistic as defined by Compusyn (www.combosyn.com) (Figure 6A). The death marker cleaved PARP indicated that the combination of PD0325901 and CX-4945 caused a larger percentage of cells to enter apoptosis at 24 h as compared to equivalent doses of single agents (Figure 6B)

A xenograft study was performed to test the effectiveness of CX-4945 and PD0325901 as a combination treatment *in vivo*. However, the effect on tumor growth by the combination of CX-4945 with PD0325901 did not differ from mice treated with PD0325901 alone (Figure 6C). All mice treated with PD0325901 or PD0325901 with CX-4945 survived until day 24 (Figure 6D). Thus, while the *in vitro* results of combining CX-4945 and PD0325901 seemed promising, the *in vivo* combination treatment was no more effective than PD0325901 alone.

## DISCUSSION

In this study we demonstrate that CK2α is overexpressed in MPNST cells and that inhibition of CK2 through shRNA or with CX-4945 causes MPNST cells to arrest in the G2/M phase of the cell cycle and undergo apoptotic cell death. β-catenin plays a significant role in MPNST cell survival *in vitro*, and we find that CK2 controls β-catenin protein stability in MPNST. Additionally, CK2 may regulate survival of MPNSTs through TCF8. CX-4945 inhibited tumor growth and increased survival in a MPNST xenograft model. It has been reported that there is minimal toxicity of CK2 inhibition in phase I clinical trials [72], making CX-4945, or other CK2 inhibitors, an appealing therapeutic approach for treating MPNSTs.

At least part of the effect of CX-4945 on MPNST cell survival is due to its reduction of β-catenin; partial rescue of CX-4945 cytotoxicity was achieved by re-expression of β-catenin using GSK-3β inhibitors, and knockdown of β-catenin phenocopies the apoptosis caused by CK2 inhibition. However, shCTNNB1 did not phenocopy the modest G2/M arrest demonstrated by CK2 inhibition with CX-4945. These results compelled us to explore other pathways. We identified TCF8 as another potential MPNST survival factor. *TCF8* is consistently overexpressed in human MPNST cells and tumors (Miller et al., 2009), and CK2 inhibition caused a decrease in TCF8 protein. Knockdown of TCF8 using shRNA induced apoptosis in MPNSTs. Thus, CK2 regulates β-catenin and TCF8 stability to promote MPNST survival. This may be a general phenomenon, as CK2α overexpression also resulted in TCF8 overexpression in breast cancer [73]. Precisely how CK2 facilitates cell cycle progression is unknown. Correlative data suggests that one CK2 substrate relevant to cell cycle regulation is SIX1 [74]. It is therefore of interest that several SIX transcripts (*SIX1- 4*) show elevated expression in MPNST (Miller et al., 2010). Although SIX1 expression was not reduced by CK2 inhibition, the potential phosphorylation of SIX1 by CK2 may be important for MPNST cell viability.

Bian et al. (2015) reported that MEK inhibition sensitizes head and neck cancer cells to the cytotoxic effects of CK2 inhibition with CX-4945 [71]. We reproduced these results in MPNST cells *in vitro*, finding that MPNST cell survival was decreased by the combination of CX-4945 and PD0325901. Our xenograft studies confirmed that PD0325901 is effective at slowing MPNST growth, as previously shown using a dose of 10 mg/kg in another xenograft model [16]. The lower dose of 1.5 mg/kg used in this study is the mouse counterpart of the current recommended clinical dose. We found that 1.5 mg/kg PD0325901 dose significantly slowed tumor growth in a xenograft model and significantly down regulated MEK signaling. However, we failed to find any added benefit of the combination in the S462TY xenograft model at the doses tested. In mice, CX-4945 has a 5 h half-life and only 20% bioavailability (61), and reduced β-catenin protein partially and transiently. Most studies using CX-4945 in xenografts have dosed mice at 75 mg/kg b.i.d., as we did [71, 62, 63, 65, 75, 61]. Under these conditions, CX-4945 is under its IC50 for prolonged periods, even when dosing twice daily. In humans, however, CX-4945 has a half-life of 25 h and thus should be more effective than in mice. Reports from an adult phase I clinical trial concluded that CX-4945 4 times daily was more effective in maintaining CK2 inhibition in blood cells than a split dose of CX-4945 twice daily [76]. Further optimization of dose or schedule could also enhance the combinatorial effect with CX-4945. In addition, new generations of CK2 inhibitors might be used to test single agent activity and identify more effective combinations.

In conclusion, inhibiting CK2 activity decreased MPNST cell survival, which correlated with a reduction in β-catenin and TCF8 protein expression. Based on these findings, CK2 inhibition enables targeting of survival pathways in MPNST and supports further investigation of CX-4945 as a potential therapeutic.

## MATERIALS AND METHODS

### Viral infection

MPNST cells were seeded at 1.5 × 10^5^ in 6 well plates and then transduced with lentivirus when wells became 10–20% confluent. Target MOI for all infections was 10. Incubation of the virus was carried out at a minimal volume of 1 ml with polybrene (8 μg/mL) (Sigma) overnight. Cells were then incubated in DMEM containing 10% FBS and 1% penicillin/streptomycin with Puromycin (2 ug/ml). Lentiviruses encoding shRNA targeting CK2 (TRCN0000000607, TRCN0000320858), β-catenin (TRCN0000314921, TRCN0000314991), and TCF8 (TRCN0000017567, TRCN0000017564) were acquired from Sigma. Sigma non-targeting lentivirus (SHC016H) was used as a control.

### RNA preparation and real time quantitative RT-PCR (qPCR)

The RNeasy kit (Qiagen) was used to isolate RNA. RNA was converted to cDNA using the ABI High capacity archive kit. Real time quantitative reverse transcriptase PCR (qPCR) was performed using Thermo scientific SYBR-green master mix. QPCR results were replicated with 3 different experimental samples and each qPCR was performed in triplicate. Expression of each gene was normalized to β-Actin. Human primers included: CSNK2A1 F-GCTGGGGGTAAGACCTTGTT and R - TTGTCTGTGTGAGCAGAGGG , β-ACTIN F-GTTGTCGACGACGAGCG and R-GCACAGAGCC TCGCCTT, AXIN2 F-CTGGTGCAAAGACATAGCCA and R-AGTGTGAGGTCCACGGAAAC, LEF1 F- CACTGTAAGTGATGAGGGG and R-TGGATCTC TTTCTCCACCCA, C-MYC R-GACAAATGAACACAG CCCAA and L- GAGTCCATGGCCAGAAAACT, CTNNB1 F- ATTGTCCACGCTGGATTTTC and R- TCGAGGACGGTCGGACT.

### Immunohistochemistry

Paraffin sections were deparaffinized, hydrated and transferred to 0.1 M citrate buffer (pH 6.0) for antigen retrieval. Slides were boiled for 10 minutes in citrate buffer, cooled at room temperature for 30 minutes, rinsed in water twice and in PBS 3 times. Sections were quenched with 3% hydrogen peroxide for 10 minutes, rinsed in PBS, and blocked in 10% normal goat serum with 0.3% Triton-X-100. Sections were incubated overnight in primary antibody diluted in block; rabbit anti CK2α on human MPNST and normal peripheral nerve (Abcam, ab76040 at a dilution of 1:200) and rabbit β-catenin (Cell Signaling, 8480 at a dilution of 1:200). Sections were then washed and incubated in goat anti rabbit biotinylated secondary antibodies (Vector, BA-1000) for 1 hour at room temperature, incubated in ABC (Vector, PK-6100) followed DAB (Vector, SK-4100) staining. Some sections were counterstained with Harris hemotoxylin. All microscopic images were acquired with Openlab software suites on a Zeiss Axiovert 200.

### Immunoblot

Cell lysates were made with radioimmunoprecipitation assay buffer (RIPA) and western blotting was performed. Membranes were probed with antibodies for CK2α 1:5000 (Cell Signaling, 2656), phosphorylated CK2 substrate 1:10,000 (Cell Signaling, 8738), PARP 1:5000 (Cell Signaling, 9542), β-catenin 1:10,000 (Cell Signaling, 9562), HRP conjugated β-Actin 1:50,000 (Cell Signaling, 5125), or TCF8 (Cell Signaling, 3396). Horseradish peroxidase-conjugated secondary antibodies (Jackson Labs) were incubated for 1 h at room temperature. Blot development was performed with ECL Plus developing system (Amersham Biosciences).

### Cell viability assays

MPNST and iHSC cell lines (500 cells/well) were seeded in triplicate in 96-well plates. Cells were selected in Puro for 48 h and absorbance was read day 3 post-infection of CK2, TCF8, or β-catenin shRNA or day 3-post treatment with CX-4945. Absorbance reagent CellTiter 96^®^ Aqueous One Solution Cell Proliferation Assay (Promega) was used. Combination drug studies using CX-4945 and PD0325901 were completed after a 72 h treatment time and cells were quantified with Biorad TC20 automated cell counter. Combination index was calculated using ComboSyn developed by Tin-Chao Chou.

### Cell cycle analysis

Approximately 200,000 MPNST cells were seeded in a six well plate and then treated with CX-4945, DMSO, or shRNAs for 24 h. Cells were harvested and fixed in methanol at −20°C for 30 minutes to overnight and then washed in PBS before being stained with propidium iodide at 50 ug/ml (Sigma). A FACSCantos was used for Flow and data was analyzed using FlowJo software.

### Mouse xenograft

1.5 × 10^6^ MPNST S462-TY cells suspended in Matrigel (BD) were injected subcutaneously into 6 to 8-week-old female athymic nude (nu/nu) mice (Harlan). Treatment of tumors began when the average tumor size was 250 mm^3^. CX-4945 sodium salt (Medchem Express, HY-50855B) was dissolved in 25 mM sodium phosphate buffer (pH = 4.3) (Sigma, 79629) and mice were treated twice daily at a dose of 75 mg/kg (71). PD0325901 was dissolved in 0.5% methylcellulose/0.2% tween 80 in water and administered once a day at a dose of 1.5 mg/kg by oral gavage. Tumor volumes were measured every 3 days.

### Cell lines

The ST88-14, S462TY, and 88-3 MPNST cell lines derived from patients with NF1 mutations. The STS26T MPNST cell line is derived from a sporadic MPNST with two WT NF1 alleles. The immortalized human Schwann cell line (iHSC) is derived from normal human sciatic nerve. The iHSCs contain no mutations in the NF1 alleles and was immortalized through expression of hTERT and CDK4^R24C^ (Dr. Margaret Wallace, manuscript in preparation). All MPNST cell lines and iHSCs were cultured in Dulbecco's Modified Eagle Medium (DMEM) containing 10% FBS and 1% penicillin/streptomycin (Fisher). Normal human Schwann cells (NHSCs) were obtained from autopsy specimens and maintained as described [77].
